# Thymosin β10 promotes tumor-associated macrophages M2 conversion and proliferation via the PI3K/Akt pathway in lung adenocarcinoma

**DOI:** 10.1186/s12931-020-01587-7

**Published:** 2020-12-22

**Authors:** Jun Zeng, Xianggui Yang, Li Yang, Wancheng Li, Yaxin Zheng

**Affiliations:** 1 Division of Pulmonary and Critical Care Medicine, Clinical Medical College and The First Affiliated Hospital of Chengdu Medical College, Chengdu, 610500 China; 2 School of Pharmacy, Key Laboratory of Sichuan Province for Specific Structure of Small Molecule Drugs, Chengdu Medical College, Chengdu, 610500 China; 3 Department of Laboratory Medicine, Clinical Medical College and The First Affiliated Hospital of Chengdu Medical College, Chengdu, 610500 China

**Keywords:** Thymosin β10, Macrophages, Proliferation, Phenotype conversion, PI3K/Akt, Lung adenocarcinoma

## Abstract

**Background:**

Thymosin β10 (TMSB10) has been reported to play a protumorigenic role in a majority of solid cancers. However, the existence of TMSB10 in immune microenvironment may contribute to the pathogenesis of lung adenocarcinoma has not been previously explored.

**Method:**

TAMs-associated TMSB10 expression was evaluated by immunohistochemistry (IHC) in 184 lung adenocarcinomas. Xenograft mice model was established to investigate the effect of TMSB10 shRNA on TAMs phenotypes. The macrophages phenotype associated cytokines IL-6, IL-8, IL-12 and TNF-α were detected by ELISA after treated with TMSB10 shRNA or scramble. Furthermore, the target proteins were detected by immunoblotting.

**Results:**

We found that high TAMs-associated TMSB10 expression was significantly correlated with the advanced TNM stage and T3/T4 tumor size. And high TAMs-associated TMSB10 expression was significantly correlated with poor overall and progression-free survival of lung adenocarcinoma, acting as an independent prognostic factor for lung adenocarcinoma. Furthermore, we investigated the biological functions of TMSB10 in macrophages in vivo and in vitro. TMSB10 knockdown dramatically reduced TAMs, THP-1 and RAW264.7 cell proliferation, and promoted macrophages phenotype conversion of M2 to M1, and TMSB10 knockdown reduced the levels of p-Akt (Sec473), p-mTOR (Sec2448) and p-p70S6K (Thr389) without effect on Akt, mTOR and p70S6K expression.

**Conclusions:**

These results demonstrate that TAMs-associated TMSB10 promotes tumor growth through increasing TAMs M2 conversion and proliferation via PI3K/Akt signaling pathway, providing a promising tumor biomarker for predicting prognosis and a potential therapeutic target for lung adenocarcinoma.

## Background

Although lung cancer morbidity shows a continuous tendency to decline in recent years, yet lung cancer accounts for a quarter of all cancer deaths worldwide [1]. Even these patients with stage I and II non-small cell lung cancer (NSCLC) received radical resection, nonsurgical approaches such as stereotactic body radiotherapy (SBRT) or Radiofrequency ablation, postoperative recurrence rate reach to over 30% in patients with early stage NSCLC [2, 3]. For stage III NSCLC, the multimodality therapies including surgical removal, chemotherapy, radiotherapy or targeted therapy, is preferable in the subsets of patients [4]. The number of postoperative patients that could benefit from the adjuvant therapies is limited [5, 6]. For lung adenocarcinoma is the most prevalent subtype of lung cancer, it is urgent to develop new treatment strategies for those recurrent patients. In recent years, the great value of immunotherapy in anticancer treatment has been confirmed, which accelerate the exploration of diverse effective immunotherapeutic strategies for various malignancies. The immune checkpoints such as cytotoxic T-lymphocyte antigen-4 (CTLA-4), programmed cell death 1 (PD-1) and programmed death ligand-1 (PD-L1) were the most promising targets, meanwhile their immunosuppressants have been clinically demonstrated effective in NSCLC, melanoma and bladder cancer et al. [7–12]. However, acquired resistance to immunotherapies was unavoidable after receiving immune checkpoints blockade [13, 14].

Tumor-associated macrophages (TAMs) are the major component of the tumor immune microenvironment in solid tumors, which help tumor cells to escape immune surveillance by recruiting immunosuppressors like regulatory T cells (Tregs) and myeloid-derived suppressor cells (MDSCs) [15]. Since TAMs are mainly consisted by antitumorigenic M1 phenotype macrophages and protumorigenic M2 phenotype macrophages, studies try to restore the antitumorigenic talents of macrophages and repress protumorigenic TAMs are prevailing in recent years [15]. Manipulating these arsenals of the innate immunity combined with known curative immunotherapies that activate the adaptive immunity looks more promising than either therapy alone.

Thymosin β10 (TMSB10) belongs to the family of β-thymosins which contain highly conserved amino acid sequence [16]. TMSB10 mainly serve as actin sequestering proteins to inhibit the formation of F-actin. TMSB10 also has been proven to be overexpressed in most human solid tumors, and regulate cancer cell proliferation and metastasis [17–21]. Notably, Yonit Lavin et al. reported that TMSB10 mRNA was unregulated in TAMs of early lung adenocarcinoma compared with those in mononuclear macrophages of adjacent normal lung tissues and peripheral blood [22].

However, the relation between TMSB10 and TAMs remains unclear, which needed to be furtherly clarified. To the best of our knowledge, this is the first study to explore the role of TMSB10 in early lung adenocarcinoma through analyzing the relation between its clinicopathological characteristics and TAMs-associated TMSB10. Moreover, we conducted the experiments in vitro and in vivo to investigate the biological effect of TMSB10 on TAMs phenotype.

## Methods

### Clinical data

The clinicopathological and survival data were obtained from patients with lung adenocarcinoma who had undergone surgery between January 2013 and December 2019. Tumors were classified according to the 8th edition of the AJCC TNM system for lung cancer. The inclusion criteria were as follows: (1) primary lung adenocarcinoma diagnosed by pathological diagnosis; (2) cases had received no previous treatment before operation. The age, gender, smoking history, tumor site, tumor size, lymphatic metastasis, TNM stage, and survival information were acquired from medical records. This retrospective study was approved by the ethics committee of our hospital, and the informed consent was exempted.

### Animal models

The mouse adenocarcinoma cell line (LLC) was purchased from ATCC (Manassas, USA). LLC were cultured in DMEM supplemented with 10% FBS. All C57BL/6 mice were obtained from the vital river company (Beijing, China). For tumor growth experiments, 2 × 10^6^ LLC cells were suspended in 100ul of DMEM and injected subcutaneously into the right back of 6 weeks old male mice. Mice were randomly divided into experimental group or control (scramble) group matched for the tumor volumes on day 4 post tumor inoculation. The experimental group mice were injected intravenously with TMSB10 shRNA lentivirus (4 × 10^9 PFU, GENE, Shanghai) every week, and scramble cohorts were injected intravenously with equivalent scramble shRNA lentivirus. Tumor sizes were measured with calipers each 5 days throughout the in vivo experiment.

### Isolation of tumor-infiltrating immune cells

The excised tumors were finely minced and suspended in HBSS (Thermo Fisher, 14,025,134) supplemented with 2%FBS (Gibco, 1921005PJ), 1% hyaluronidase (Sigma, 37326-33-3), Collagenase 4 at 1 mg/mL (Sigma, SCR103) and 0.25% DNase I (Roche, 11284932001) at 37℃ on a shaker at 80 rpm for 2 h. The mixture was then mixed with 10% PBS (suspended in HBSS) in a 1:1 ratio and filtered through a 50um mesh. Percoll (Sigma, P1644) was used to purify tumor-infiltrating leukocytes (TILs) via density gradient centrifugation. Briefly, 60% Percoll was firstly added to the bottom of a glass tube and then 30% Percoll was carefully added by a Pasteur pipette. Finally, the filtered cells were gently resuspended in the 30% Percoll. The suspension was centrifuged at 400*g* for 25 min at room temperature. After centrifugation, the pellet of erythrocytes and excess buffer/percoll were removed, leaving a purified population of leukocytes at the interface. The purified tumor-infiltrating leukocytes were washed twice with FACS buffer prior to flow cytometry analysis.

### Flow cytometry

Cell staining was operated on ice and away from light. For surface staining, the purified cells from tumor tissues were incubated with mouse Fc block (1:200, BD Biosciences, BD553141) in 4 ℃ for 30 min before staining with relevant conjugated antibodies. The relevant fluorescent-labeled antibodies were diluted in FACS buffer and applied to incubate for 30 min. For intracellular staining, cells were subsequently washed by FACS buffer and fixed (Fixation and Permeabilization Solution, BD Biosciences, BD554722), then the relevant fluorescent-labeled antibodies, diluted in Perm/Wash Buffer (BD Biosciences, BD557885), were used to incubate for 30 min. Upon staining, cells were resuspended in FACS buffer (25 mM HEPES, 2% FBS, 10 mM EDTA, 0.1% sodium azide in PBS) and passed through 50um mesh filter before flow cytometry analysis. The stained cells were run on a BD LSRFortessa X-20 Flow Cytometer equipped with FACSDiva software (BD Biosciences). FlowJo software (Treestar) was applied for analyzing flow cytometry data. Forward scatter (FSC) and side scatter (SSC) were used to gate on single nucleated cells and to eliminate cell debris and doublets.

### Immunoblotting and ELISA assay

THP-1 and RAW264.7 cell lines were cultured in RPMI-1640 medium supplemented with 10% FBS. After treatment with indicated shRNA lentivirus (MOI 10), lipopolysaccharide (LPS) (100 ng/ml) or IL-4 (15 ng/ml) for 72 h, THP-1 and RAW264.7 cells were harvested in RIPA buffer (Sigma-Aldrich) supplemented with protease inhibitors and phosphatase inhibitors (Roche). Next, equal amounts of total protein lysates (40 μg) were separated by SDS-PAGE before transferred to a PVDF microporous membrane, and stained with antibodies against TMSB10 (1:1000, Sant cruz, sc-514309), AKT (1:1000, Abcam, ab8805), p-AKT(Ser473) (a1:3000, Abcam, ab81283), mTOR (1:2000, Abcam, ab2732), p-mTOR(sec2448) (1:1000, Abcam, ab109268), p70S6K (1:2000, CST, 9202S), p-p70S6K(Thr389) (1:1000, CST, 9234T) and GAPDH (1:5000,CST, 5174S) as previously described [19]. Meanwhile, the extracellular IL-6, IL-10, IL-12 and TNF-α levels in serum-free conditioned media from THP-1 and RAW264.7 cells treated with indicated lentivirus, LPS or IL-4 for 72 h were measured using the ELISA kit (Elabscience, Wuhan).

### Immunohistochemistry

Immunohistochemical assay was performed on the 5 mm thick slides, using mouse anti-human TMSB10 (1:500, Sant cruz, sc-514309) and CD68 (1:1000, CST, 76437) primary antibody, as previously [23]. Two experienced investigators independently scored the TAMs-associated TMSB10 without knowing the clinical data. The proportion of positive macrophage was scored as 0 (0–9% positive macrophages), 1 (10–25% positive macrophages), 2 (26–50% positive macrophages) and 3 (> 50% positive macrophages) respectively. Staining intensity was scored as 0 (no staining), 1 (weak staining), 2 (moderate staining) and 3 (strong staining). The final scores ranged from 0 to 3, ≥ 2 score was defined as high TAMs-associated TMSB10 expression and < 2 score was defined as low TAMs-associated TMSB10 expression according to previous method [18].

### Cell proliferation assay

The THP-1 and RAW264.7 cells proliferation were measured by a Cell counting Kit-8 (CCK-8) kit (Beyotime, Shanghai, China). Briefly, cells were seeded into 96-well plates. After transfection of indicated shRNA lentivirus for 72 h, the WST-8 reagent was added to each well for 1 h incubation. The optical density (OD) value (450 nm) was measured using a microplate reader.

### Statistical analysis

Statistical analyses were performed by SPSS 22.0 software (IBM Corporation, NY, USA). The correlation between TMSB10 expression and clinical pathological variables were investigated by Chi-square test. The Kaplan–Meier method and Cox proportional hazard model were used to investigate the prognostic factors for progression free survival (PFS) and overall survival (OS). Unpaired two-tailed Student’s t tests and/or analysis of variance (ANOVA) were used to calculate the significance. *P* < 0.05 was considered significant unless otherwise noted.

## Results

### High TAMs-associated TMSB10 is correlated with clinicopathologic characteristics of lung adenocarcinoma

A total 257 cases with lung adenocarcinoma were preliminary enrolled in our study. 73 cases of them were initially excluded: (1) 65 patients had received systemic therapy before enrollment and (2) 8 cases had other malignancies or multiple primary tumors. Finally, 184 cases were enrolled in this study. The clinicopathologic features of the enrolled 184 cases were presented in Table 1. The average age of diagnosis was 63 years (age from 35 to 86 years) with mean follow-up of 37 months (range from 4 to 83 months). Of the whole patients, 44.6% were female, 49.5% were non-smokers, 64.1% were at stage 1 + II and 35.9% were at stage III, 114 patients (62.0%) showed no lymphatic metastasis.

**Table 1:** The Correlation between TAMs-associated TMSB10 expression and clinicopathological characteristics of lung adenocarcinoma

Variables	All, N (%)	TAMs-associated TMSB10 expression		*P*
		Low, N (%)	Hihg, N (%)	
Age, mean ± SD	63.0 ± 9.9	59.2 ± 8.1	64.7 ± 10.1	< 0.001
< 65	102 (55.4)	43 (75.4)	59 (46.5)	< 0.001
≥ 65	82 (44.6)	14 (24.6))	68 (53.5)	
Gender				
Male	102 (55.4)	30 (51.7)	72 (57.1)	0.608
Female	82 (44.6)	28 (48.3)	54 (42.9)	
Smoking history				
Smoker	93 (50.5)	29 (50.9)	64 (50.4)	0.952
Non-smoker	91 (49.5)	28 (49.1)	63 (49.6)	
Tumor site				
Upper or middle	126 (68.5)	40 (70.2)	86 (67.7)	0.740
Lower	58 (31.5)	17 (29.8)	41 (32.0)	
Tumor size				
T1 + T2	158 (85.9)	43 (75.4)	115 (90.6)	0.007
T3 + T4	26 (14.1)	14 (24.6)	12 (9.4)	
Lymphatic metastasis				
N0	114 (62.0)	35 (60.3)	79 (62.7)	0.666
N1–N3	70 (38.0)	23 (39.7)	47 (37.3)	
TNM stage				
I + II	118 (64.1)	42 (73.7)	76 (59.8)	0.036
III	66 (35.9)	15 (26.3)	51 (40.2)	

**Fig. 1:**
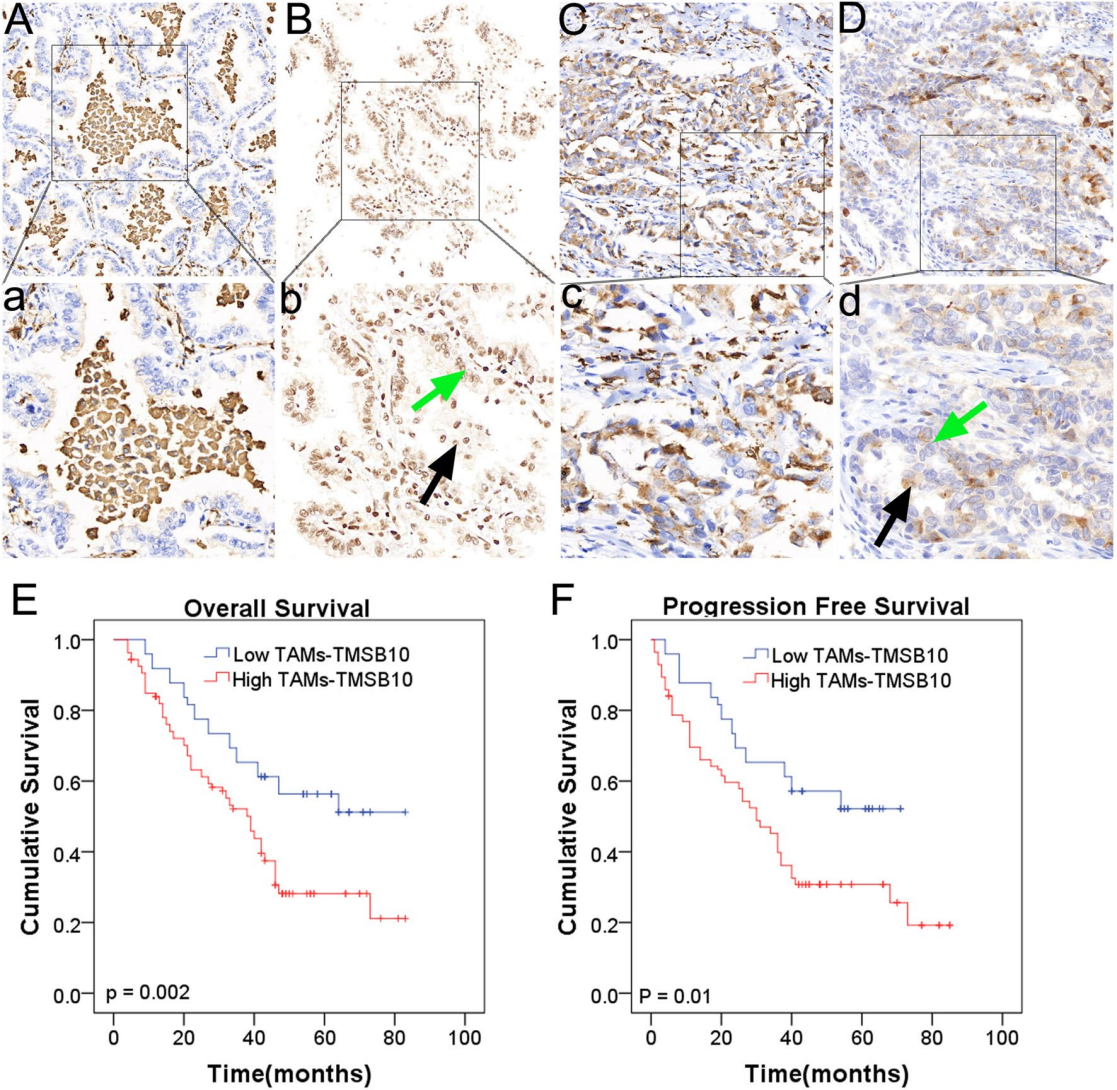
High TAMs-associated TMSB10 expression is correlated with poor prognosis for patients with early lung adenocarcinoma. **a**–**d** Representative IHC staining for CD68 and high TMSB10, CD68 and low TMSB10, respectively. Up panel shows 100 × , down panel shows 200 × . **e**, **f** The association between TAMs-associated TMSB10 expression and Progression free survival and overall survival of patients with early lung adenocarcinoma. Black arrow indicates macrophages, and green arrow indicates cancer cells

As TAMs is crucial for lung cancer progression, those genes that promote protumorigenic M2 polarization of TAMs might promote cancer progression. We found the CD 68 protein was mainly expressed in TAMs, and TMSB10 protein was expressed in both tumor cells and TAMs (Fig. 1a–d). High TAMs-associated TMSB10 expression preferred to be detected in older patients (*P* < 0.001). High TAMs-associated TMSB10 expression was significantly associated with advanced TNM stage (*P* = 0.036) and T3/T4 tumor size (*P* = 0.007) (Table 1), indicating TAMs-associated TMSB10 might contribute to tumor proliferation or metastasis. There are no statistically differences in the relation between TAMs-associated TMSB10 expression and other clinicopathologic characteristics.

### High TAMs-associated TMSB10 is correlated with the poor prognosis of lung adenocarcinoma

To investigate the prognostic value of TAMs-associated TMSB10 in patients with lung adenocarcinoma, we had collected follow-up data and performed survival analysis. Kaplan–Meier analysis showed that patients with low TAMs-associated TMSB10 expression had significantly better PFS (5-year survival rate: 53.0% versus 30.1%, *P* = 0.002) as well as better OS (5-year survival rate: 57.0% versus 25.0%, *P* < 0.001) compared with those with high TAMs-associated TMSB10 expression (Fig. 1e, f).

The Cox regression analysis of PFS showed that high TAMs-associated TMSB10 expression (HR: 1.726, 95%CI: 1.061–2.808, *P* = 0.028) might be independent risk factors of PFS. Meanwhile, it was also showed that high TAMs-associated TMSB10 expression (HR: 1.94, 95%CI: 1.189–3.167, *P* = 0.008) might be independent risk factors of OS.

### TAMs-associated TMSB10 knockdown represses tumor growth

**Fig. 2:**
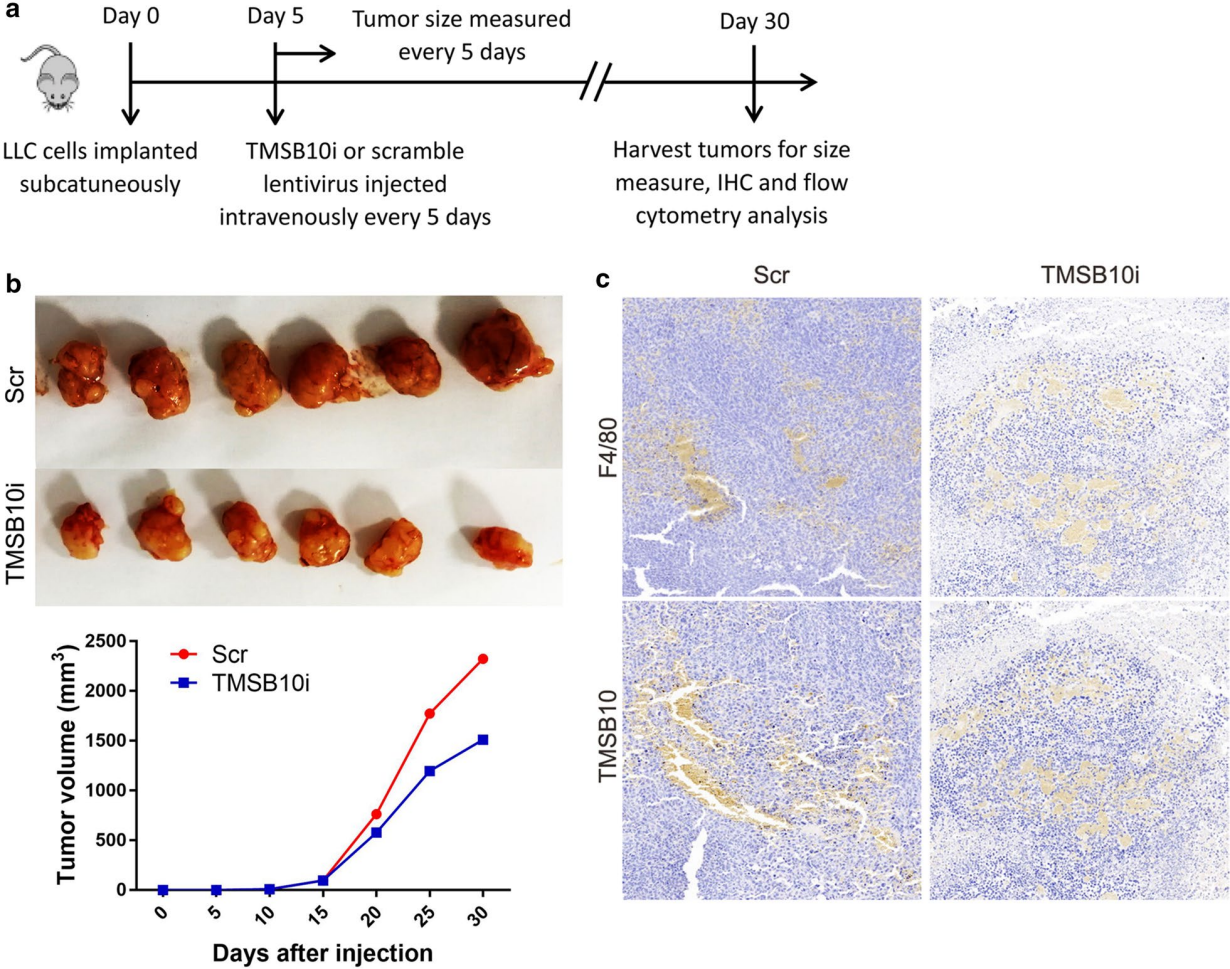
TAMs-associated TMSB10 knockdown regresses tumor growth. **a** Schema of the tumor transplantation experiment (n = 4 for each groups). **b** The tumors were excised on day 30 post shRNA lentivirus injected and tumor size was measured. 5 × 10^6^ Lewis lung cancer cells (LLC) were injected subcutaneously in the right back of mice on day 0, shRNA lentivirus injected intravenously every 5 days from day 5. **c** F4/80 and TMSB10 in TAMs are detected using IHC staining. Scr: scramble shRNA; TMSB10i: TMSB10 shRNA

To identify the effect of TAMs-associated TMSB10 on the progression of lung adenocarcinoma, we conducted a xenograft model. The LLC subcutaneously injected mice received TMSB10 shRNA and scramble shRNA lentivirus intravenously, respectively (Fig. 2a). We found that TMSB10 knockdown significantly attenuated the growth of lung adenocarcinomas (Fig. 2b. And the infiltrating TAMs and TAMs-associated TMSB10 expression reduced by TMSB10 knockdown (Fig. 2c). Therefore, we speculated that TMSB10 knockdown might repress the M2 phenotype and proliferation of macrophages.

### TMSB10 knockdown promotes macrophages M1 conversion and inhibits macrophages proliferation

**Fig. 3:**
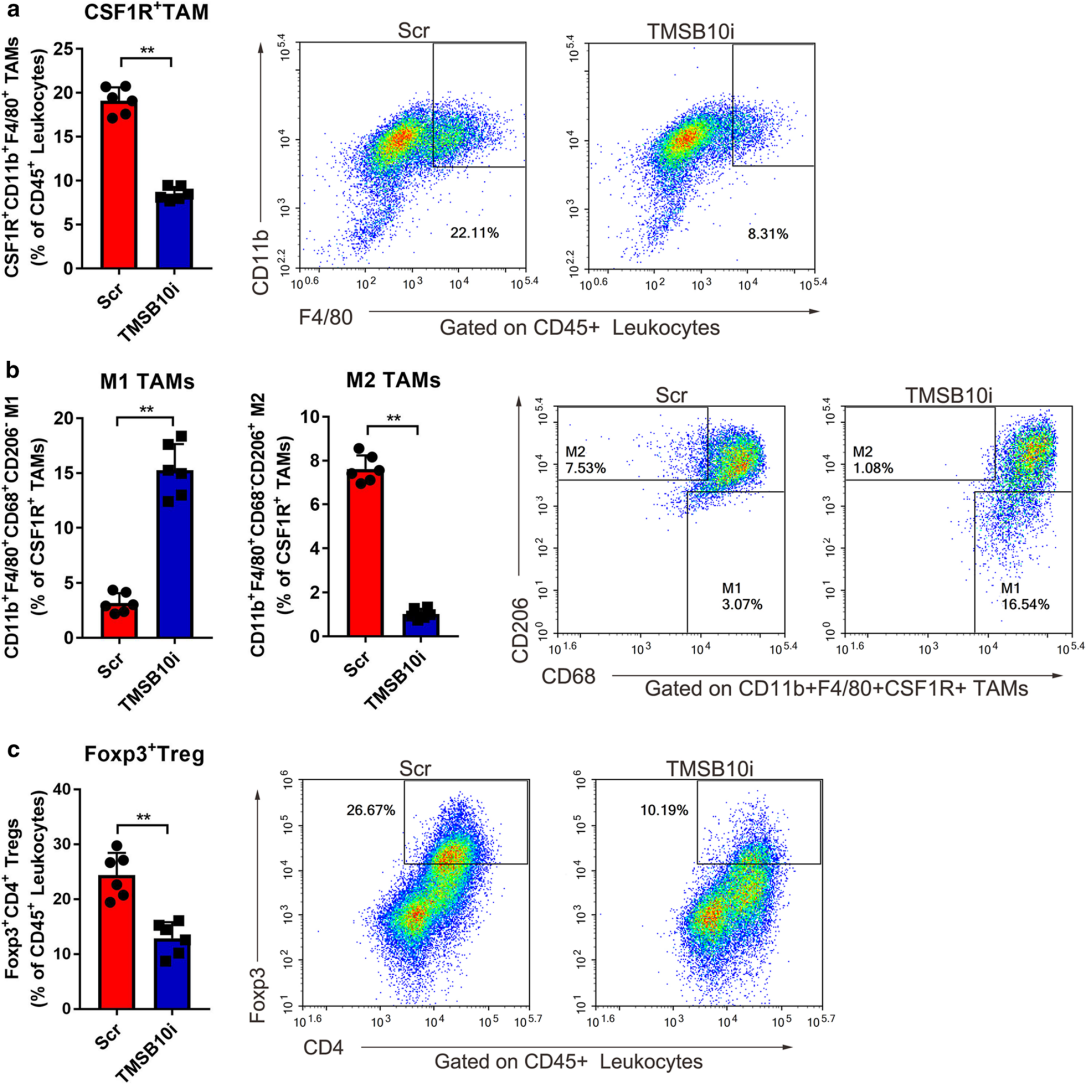
TMSB10 knockdown promotes M1 conversion of TAMs. **a** The total CSF1R^+^ TAMs, **b** M1 phenotype TAMs, M2 phenotype TAMs and **c** regulatory t cells (Tregs) of xenograft tumors were analyzed by flow cytometry on day 30 post LLC were injected subcutaneously. Scr: scramble shRNA; TMSB10i: TMSB10 shRNA. The data represents mean ± SD in each group, n = 3; **P < 0.01

We further performed flow cytometry analysis of total TAMs (CD45^+^CD11b^+^F4/80^+^CSF1R^+^), M1 phenotype TAMs (CD11b^+^F4/80^+^CSF1R^+^CD68^+^CD206^−^), M2 phenotype TAMs (CD11b^+^F4/80^+^CSF1R^+^CD68^−^CD206^+^) and Tregs (CD45^+^Foxp3^+^CD4^+^) subpopulations of LLC tumors derived from xenograft mice. And we found that the proportion of CSF1R^+^ TAMs in CD45^+^ TILs was reduced in TMSB10 knockdown xenograft tumors (Fig. 3a). Meanwhile, the proportion of M2 TAMs in total CSF1R^+^ TAMs and the proportion of Foxp3^+^ Tregs in CD45^+^ TILs were dramatically reduced in TMSB10 knockdown group compare to those in scramble group (Fig. 3b, 3c).

**Fig. 4:**
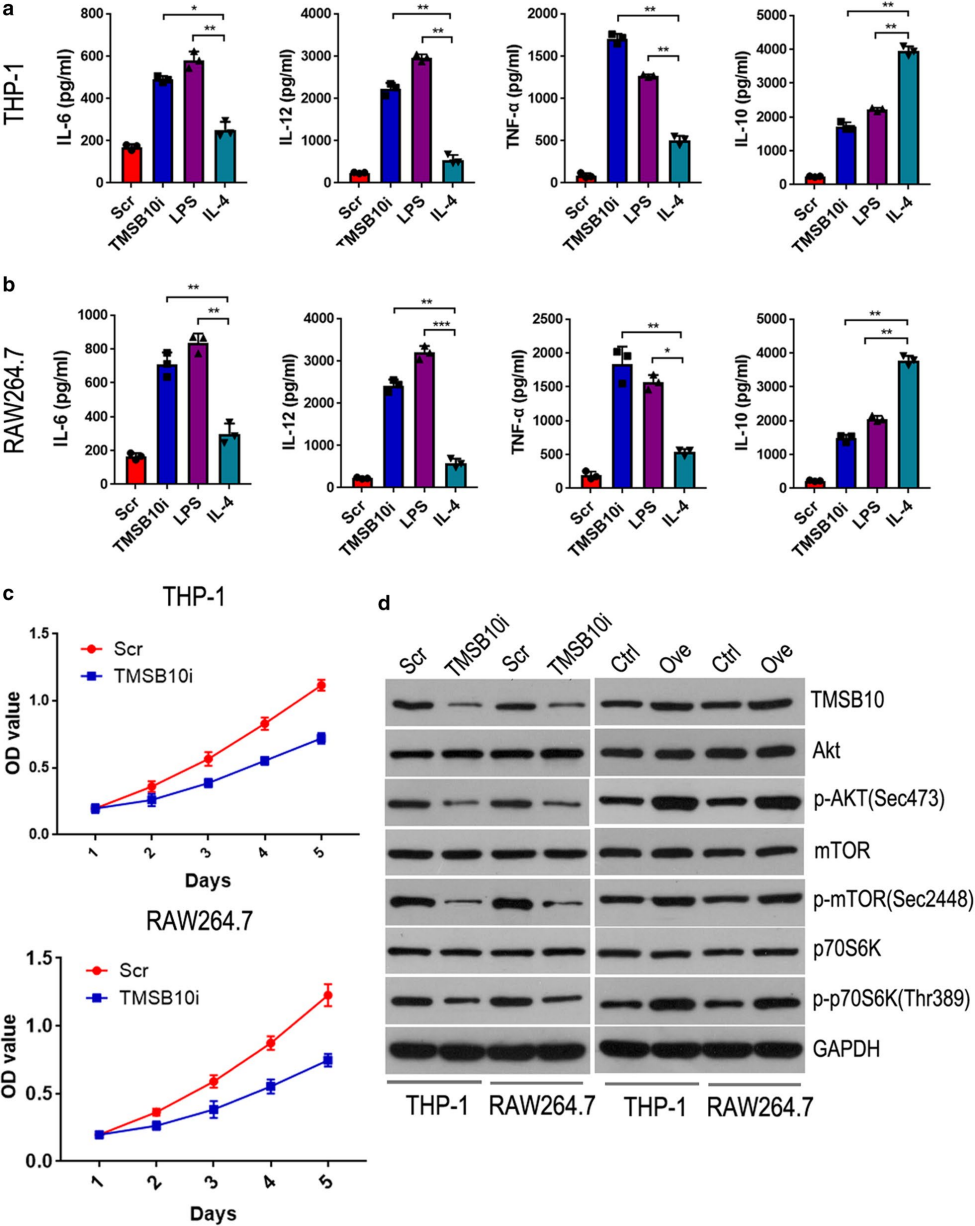
TMSB10 promotes cell proliferation and M2 conversion of THP-1 and RAW264.7 via PI3K/Akt pathway. **a** THP-1 Supernatants were collected after 72 h treatment for analysis of IL-6, IL-10 and TNF-α and IL-12 secreted level (pg/ml) by ELISA. **b** RAW264.7 Supernatants were collected after 72 h treatment for analysis of IL-6, IL-10 and TNF-α and IL-12 secreted level (pg/ml) by ELISA. **c** The proliferation of THP-1 and RAW264.7 was detected by cell proliferation assay. **d** Immunoblotting result for TMSB10, Akt, p-Akt (Sec473), mTOR, p-mTOR (Sec2448), p70S6K, p-p70S6K (Thr389) and GAPDH proteins expression in THP-1 and RAW264.7 cells. Scr: scramble shRNA; TMSB10i: TMSB10 shRNA; Control: vector control; OVE: TMSB10 overexpression vector. The data represents mean ± SD in each group, n = 3; *P < 0.05, **P < 0.01

We next explored the biological role of TMSB10 in macrophages in vitro. Given LPS could facilitate macrophages M1 polarization, and IL-4 could induce macrophages M2 polarization. We found the macrophage M1 markers IL-6 and IL-12 in TMSB10 knockdown group or in LPS group were secreted more than in IL-4 group, and TNF-α in TMSB10 knockdown group secreted more than in LPS group or in IL-4 group. However, the macrophage M2 marker IL-10 was secreted significantly less in TMSB10 knockdown group or in LPS group than in IL-4 group(Fig. 4a, b). Meanwhile, the proliferation of THP-1 and RAW264.7 cells was significantly inhibited by TMSB10 knockdown (*P* < 0.05) (Fig. 4c).

### *TMSB10 promotes macrophages M2 conversion and proliferation *via* PI3K/Akt pathway*

A previous study reported that PI3K/Akt signal was the downstream pathway of TMSB10 and played a key role in promoting breast cancer growth and metastasis by activating the Akt/FOXO pathway [19]. To investigated the underlying mechanisms of the biological effect of TMSB10 on macrophages, we intended to explore the PI3K/Akt signaling pathway. We detected PI3K/Akt pathway proteins in THP-1 and RAW264.7 cells stably-transfected with TMSB10 shRNA and scramble shRNA. We found that TMSB10 knockdown could reduce the levels of p-Akt (Sec473), p-mTOR (Sec2448) and p-p70S6K (Thr389), but had no effect on Akt, mTOR and p70S6K expression levels (Fig. 4d).

Furthermore, we found that TMSB10 overexpression could activate the PI3K/Akt signaling pathway by increasing the levels of p-Akt (Sec473), p-mTOR (Sec2448) and p-p70S6K (Thr389) without changing the levels of Akt, mTOR and p70S6K in THP-1 and RAW264.7 cells (Fig. 4d). Overall, these results suggested that the PI3K/Akt signaling pathway was involved in TMSB10-induced M2 phenotype conversion and proliferation of macrophages.

## Discussion

TMSB10, a 43-amino acid residues β-thymosin, is found to be overexpressed in human lung cancer, liver cancer, breast cancer, ovarian cancer, gastric cancer, pancreatic cancer, thyroid cancer and renal cell carcinoma et al. [18, 21, 24–28]. And TMSB10 mRNA is found to be overexpressed in TAMs of early lung adenocarcinoma compared with in adjacent normal lung macrophages and peripheral blood monocytes [22]. However, the clinicopathological significance of TAMs-associated TMSB10 in lung cancer remains largely unknown. We firstly carried out the investigation on the relationship between TAMs-associated TMSB10 and the clinicopathological features of lung adenocarcinoma. First, we found that high TAMs-associated TMSB10 expression was significantly associated with advanced TNM stage and bigger tumor size. Moreover, High TAMs-associated TMSB10 was significantly correlated with poor PFS and OS. Next, we conducted xenograft model to investigate the role of TMSB10 in tumors. We found that TMSB10 knockdown significantly attenuated the xenograft tumor growth, and the infiltrating TAMs and TAMs-associated TMSB10 expression was reduced by TMSB10 knockdown. Nevertheless, The biological role of TMSB10 in lung adenocarcinoma TAMs was still unknown.

Most studies showed that knockdown of TMSB10 reduced cancer cell proliferation or migration [18, 19, 29–31]. However, Lee et al. reported that overexpression of TMSB10 caused F-actin stress fibers disruption, notably reduced cell growth and increased apoptosis [24]. Zhang et al. showed that TMSB10 played a contradictory role in cell proliferation in different kind of cell [32]. Therefore, in order to investigating the role of TMSB10 in TAMs of lung adenocarcinoma, we isolated the tumor-infiltrating immune cells in xenograft tumors, and found that CSF1R^+^ TAMs and Foxp3^+^ Tregs were reduced in TMSB10 knockdown group. TMSB10 knockdown could significantly promote the M2 to M1 phenotype conversion of TAMs in vivo. Meanwhile, the proliferation of macrophages was repressed by TMSB10 knockdown. And the level of macrophage M1 markers IL-6, IL-12 and TNF-α were increased by TMSB10 knockdown, yet the level of macrophage M2 marker IL-10 was repressed by TMSB10 knockdown in vitro. Further studies are needed to elucidate the underling mechanism for its biological role.

PI3K/Akt is an important pathway for cellular growth, survival and protein synthesis. Recent studies have reported that resident macrophages can proliferate via different signaling so as to maintain cell number in type 2 immunity [33, 34]. Meanwhile, the anti-inflammatory M2 macrophage could been activated by PI3K/Akt signaling [35]. We further demonstrated that THP-1 and RAW264.7 cells could proliferate rapidly and be converted to M2 phenotype by activating TMSB10-dependent PI3K/Akt signaling pathway.

## Conclusions

In summary, these results demonstrated that TMSB10 was remarkably overexpressed in TAMs and negatively associated with the prognosis of lung adenocarcinoma, and TMSB10 knockdown dramatically repressed xenograft tumor growth in vivo. TMSB10 knockdown promoted antitumorgenic M1 conversion and repressed the proliferation of macrophages via PI3K/Akt signaling in vivo and in vitro. Taken together, our findings suggest that TAMs-associated TMSB10 promotes tumor through increasing TAMs proliferation and M2 phenotype conversion via PI3K/Akt signaling, which might be an indicator for prognosis, seems as a promising novel therapeutic target for human lung adenocarcinoma.

## Data Availability

The software, databases and all relevant raw data are freely available to scientists.

## Abbreviations

TAMs: Tumor-associated macrophages
Tregs: Regulatory T cells
NSCLC: Non-small cell lung cancer
LLC: Lewis lung cancer
MDSC: Myeloid-derived suppressor cells
FSC: Forward scatter
SSC: Side scatter
